# The role of lived and living experience in evidence-based decision-making in suicide prevention

**DOI:** 10.1093/heapro/daag139

**Published:** 2026-09-10

**Authors:** Max Tran, Jaelea Skehan, Jon Eddy, Jessica Wilcox, Lennart Reifels

**Affiliations:** Everymind, PO Box 833, Newcastle, NSW 2300, Australia; Everymind, PO Box 833, Newcastle, NSW 2300, Australia; School of Medicine and Public Health, University of Newcastle, University Dr, Newcastle, NSW 2308, Australia; Everymind Lived Experience Advisory Group, Everymind, PO Box 833, Newcastle, NSW 2300, Australia; Everymind Lived Experience Advisory Group, Everymind, PO Box 833, Newcastle, NSW 2300, Australia; Melbourne School of Population and Global Health, University of Melbourne, 207 Bouverie St, Carlton, VIC 3053, Australia

**Keywords:** lived experience, suicide prevention, evidence-based decision-making, knowledge translation

## Abstract

Evidence-based decision-making in suicide prevention is shaped not only by research evidence but also by population needs, resources, and the implementation context. While lived and living experience is a core focus of contemporary suicide prevention policy and practice in Australia, little is known about how it is valued, integrated, and reconciled with other decision-making factors. The present study explored how lived and living experience knowledge informs evidence-based decision-making in suicide prevention, how factors that affect decision-making can be reconciled, and what resources are needed to improve it. Eighty-nine participants from the Australian suicide prevention sector engaged in a qualitative, participatory workshop at the 2025 Lived Experience of Suicide Summit. Participants included people with lived and living experience of suicide and those working across policy, service delivery, funding, and research. Data were collected through structured activities and analysed using reflexive thematic analysis and descriptive content analysis. Participants perceived lived and living experience knowledge to inform a small proportion of suicide prevention decisions. Findings indicated mixed effectiveness of current engagement practices, with tokenism and disengagement being key markers of ineffective engagement. Systemic issues, such as power dynamics and sector culture, were identified as factors that impact how knowledge and expertise are valued and integrated into decision-making. Strengthening the integration of lived and living experience into decision-making requires a paradigm shift that positions lived and living experience knowledge as a legitimate and integral form of evidence. This includes resources to support embedding engagement into suicide prevention approaches, structural supports, and increased implementation capacity.

Contribution to Health Promotion

- This paper improves understanding of how lived and living experience knowledge informs evidence-based decision-making in suicide prevention.
- Findings suggest that lived and living experience currently informs a limited proportion of decisions, with tokenism, power imbalances, and sector culture affecting how this knowledge is used.
- Decisions are stronger when lived and living experience knowledge is valued alongside research evidence, population needs, and the implementation context.
- Effective integration of lived and living experience requires practical enablers, including shared decision-making authority, fair remuneration, clear governance, leadership commitment, and sufficient time and resources to support meaningful involvement.

The challenges associated with translating evidence into practice are well established in the literature. Different disciplines have developed unique approaches for defining and using evidence-based practice, while continuing to experience shared challenges, including how evidence is defined and weighted relative to other considerations such as professional expertise, available resources, and the perspectives of populations for whom interventions are designed (Satterfield *et al*. 2009). These challenges highlight that evidence-based decision-making is not a straightforward or linear process, but is complex, context-dependent, and value-laden. In response, Satterfield *et al*. proposed a trans-disciplinary model of evidence-based practice that positions decision-making at the intersection of the best available research evidence, population needs, and available resources, all within the broader environmental and organizational context in which decisions are made (Satterfield *et al*. 2009).

As suicide prevention spans multiple disciplines, examining evidence-based decision-making through a transdisciplinary lens may support conceptualization beyond empirical evidence alone. There is growing recognition that decisions in suicide prevention are rarely shaped solely by the effectiveness of interventions (Reifels *et al*. 2022). Instead, decisions are influenced by the needs and preferences of a particular population, the available funding and workforce, and the broader system or community context in which interventions are implemented (Reifels *et al*. 2022, Krishnamoorthy *et al*. 2025b). As a result, interventions with limited empirical evidence may still be implemented, whereas interventions with strong research support may not be adopted if contextual or population factors are unfavourable (Reifels *et al*. 2022).

Involving the intended beneficiaries of an intervention in the design and implementation has a long-standing history. Participation frameworks conceptualize engagement across a spectrum, ranging from minimal or tokenistic involvement to shared or empowered decision-making. Arnstein’s (1969) ladder of participation was an early example supporting citizen participation, defined as the redistribution of power to deliberately include individuals who are excluded from decision-making. The International Association for Public Participation (IAP2) Public Participation Spectrum is also widely used to guide strategies for engaging individuals in decision-making processes (IAP2 2018) and has been specifically adapted to guide lived experience involvement in suicide prevention (Roses in the Ocean 2022a). The term *lived and living experience* of suicide is used to capture a range of past, current, and future experiences. This includes experiences of suicidal thoughts, surviving a suicide attempt, supporting a loved one experiencing suicidal distress, or being bereaved by suicide (IASP 2017, Roses in the Ocean 2022b). In this paper, the term *lived experience* will be used for brevity. The knowledge and skills derived from a person’s lived experience are referred to as *lived experience knowledge*. The term *lived experience expertise* describes the unique combination of individual and collectively derived experiential knowledge that can be used to benefit others (Byrne and Roennfeldt 2025).

Integrating lived experience into suicide prevention approaches is recognized as an enabler of implementation success (Kasal *et al*. 2023, Krishnamoorthy *et al*. 2025b); yet doing so is not without challenges or resistance. Lived experience engagement was identified as a key domain in an international Delphi expert consensus study focused on developing guidelines for applying implementation science in suicide prevention (Krishnamoorthy *et al*. 2025a). Despite broad consensus, there was considerable variation in responses within the expert panel, with some expressing resistance to the idea of lived experience integration, considering it a ‘fad’, stigmatizing, risky, or impractical, while others had engaged people with lived experience and observed positive outcomes. Nonetheless, the value of including people with a lived experience in suicide prevention is strongly reflected in Australian suicide prevention policy (National Suicide Prevention Adviser 2020, NSPO 2025a, 2025b). A growing body of literature has explored integrating lived experience into suicide prevention research (Krysinska *et al*. 2023, 2025, Webb *et al*. 2024), interventions (Watling *et al*. 2022), the workforce (Hawgood *et al*. 2023), and general suicide prevention activities (Wayland *et al*. 2020, Purdon *et al*. 2025). Further, several resources and tools exist for integrating lived experience in research (The ALIVE National Centre for Mental Health Research Translation 2024), service guidelines (Roses in the Ocean and Folk 2023a, 2023b, 2023c), and broader suicide prevention activities (Roses in the Ocean 2022c). The policy developments, literature, and resources in the Australian suicide prevention sector reflect broader movements towards public and patient involvement in health research (Greenhalgh *et al*. 2019), health policy (Baumann and Brütt 2021), and healthcare (Ocloo and Matthews 2016).

Despite this growing emphasis on lived experience knowledge, guidance on how lived experience knowledge is integrated into decision-making remains limited (Krysinska *et al*. 2023, Krishnamoorthy *et al*. 2025a). Lived experience is often positioned as complementary to evidence rather than a type of evidence, reinforcing assumptions that experiential knowledge lacks methodological rigour (Madden and Speed 2017, Roses in the Ocean 2025). This framing overlooks the ways lived experience can inform priority-setting, service design, and the interpretation and implementation of research findings (Madden and Speed 2017, Roses in the Ocean 2025). To date, little empirical evidence has examined how lived experience knowledge is valued, reconciled, and used alongside other forms of evidence in suicide prevention.

## The current study

Despite the transdisciplinary model, evidence-based decision-making is often oversimplified, with an overemphasis on quantitative evidence. This narrow focus overlooks factors such as lived experience knowledge and the implementation context, both key to developing approaches that are effective and adaptable. While numerous resources exist to support lived experience involvement in suicide prevention approaches, there remains a lack of empirical research examining *how* to integrate lived experience knowledge into decision-making. To our knowledge, no published studies have explicitly looked at the role of lived experience in suicide prevention decision-making. A workshop at the Lived Experience of Suicide Summit in Brisbane in March 2025 provided an opportunity to examine evidence-based decision-making in the Australian suicide prevention sector. This study addressed the following questions:

1. How does lived experience knowledge inform evidence-based decision-making?
2. How can factors that impact decision-making be best reconciled?
3. What tools or resources could be used to improve evidence-informed practice in suicide prevention?

## Methods

### Design

This study used an exploratory qualitative design, employing participatory workshop methods to examine the role of lived experience in evidence-based decision-making in suicide prevention. An interactive workshop titled ‘Evidence-based decision-making in suicide prevention: The role of lived experience’ was held in Brisbane on 14 March 2025 as part of the biennial Lived Experience of Suicide Summit hosted by Roses in the Ocean, Australia’s national lived experience of suicide organization.

The workshop was attended by conference delegates, including people with a lived experience of suicide and members of the suicide prevention sector, ranging from government, community, and non-government organizations, clinicians, corporate organizations, and researchers (noting people likely had multiple roles). The workshop was planned by all authors and co-facilitated by J.S., J.E., J.W., and L.R. Two co-authors (J.E. and J.W.) contributed as Lived Experience Advisory Group members. The primary aim of the workshop was to explore how lived experience knowledge informs evidence-based decision-making in suicide prevention and inform the development of tools and resources to support improved practice.

Workshops are an established qualitative and participatory method for engaging diverse stakeholders and generating practice-relevant insights, particularly in complex social policy contexts (Löhr *et al*. 2020, O’Cathain 2025, Trettin *et al*. 2026). The 2025 Lived Experience of Suicide Summit presented a practical opportunity to explore the perspectives of those with lived experience of suicide, alongside people working in the suicide prevention sector. The methodological approach and data collection processes were planned *a priori* to support ethical and methodologically sound research.

Ethical approval was obtained in advance from the Hunter New England Research Ethics Committee (Reference 2025/ETH00166) to publish workshop findings, create a summary report for participants, and develop a peer-reviewed publication.

### Participant recruitment

Participants were Lived Experience of Suicide Summit attendees who read and agreed to a ‘Readiness to be Involved’ statement during the registration process. This ensured that attendees, many of whom have a lived experience of suicide, were both cognisant of the Summit’s purpose and in a psychologically safe position to attend and contribute.

At the commencement of the workshop, facilitators distributed written information outlining the research and implied consent. Participants were informed that workshop responses would be analysed and summarized to inform the development of resources, a workshop summary report, and a research publication. Participants were advised that they could request that their contributions be excluded at any point by informing the facilitator or scribe at their table. No participants requested that their contributions be withdrawn.

### Data collection

Participant demographic information was collected during Summit registration, with attendees able to select multiple options, including being Aboriginal and Torres Strait Islander, culturally and linguistically diverse, LGBTIQA+, or living with a disability.

A brief warm-up activity was conducted, where participants responded individually, in writing, to the following question: To what degree do you think people with lived and living experience are involved in the decision-making process in suicide prevention (as a percentage)?

Activity 1 used a World Café format, a participatory method designed to facilitate discussion of critical questions with large groups (Löhr *et al*. 2020). Three stations were established to represent different decision-making settings: (i) policy and strategy; (ii) service design and delivery; and (iii) funding and priority-setting. Two pieces of paper were placed next to each other with the anchors: ‘Less effective’ and ‘More effective’. Participants selected a sticky note colour, which identified their primary experience based on three choices: (i) lived or living experience of suicidal thoughts, distress, and suicide attempt; (ii) lived or living experience as a family, friend, carer, or person bereaved by suicide; and (iii) someone working in suicide prevention. Participants who identified with multiple perspectives were able to choose which group best reflected how they wished their input to be recorded for the workshop.

Activity 2 involved roundtable discussions organized by decision-making settings: (i) policy and strategy; (ii) service design and delivery, or (iii) funding and priority-setting. Each table nominated a scribe and responded to three structured questions written on paper: (i) What makes some strategies more effective or less effective? (ii) How can we combine lived and living experience and other types of evidence to make better decisions in the future? (iii) What tools or resources would be needed to make each idea possible? All written workshop outputs (e.g. sticky notes, worksheets, and table papers) were collected and transcribed verbatim for analysis.

### Data analysis

All data collected were de-identified prior to analysis, with responses not attributable to individual participants. Data were transcribed and analysed by M.T., with constructed themes discussed and revised with J.S., J.E., J.W., and L.R. to support reflexive interpretation and analytic depth.

Different analytic approaches were used across the workshop activities, reflecting the nature of the data generated and the specific research questions addressed. Activity 1 generated discursive data exploring how lived experience informs decision-making and was therefore analysed using reflexive thematic analysis, recognizing the positionality of the researchers and the influence of research, practice, and experiential perspectives on interpretation (Braun and Clarke 2006). Interpretation was guided by the authors’ experiences as public health practitioners (M.T., J.S.), mental health and suicide prevention researchers (M.T., J.S., L.R.), and lived and living experience advisors (J.E., J.W.). The authors bring a variety of lived and living experience to the work, including experiences of suicidal distress, bereavement, and supporting a loved one (J.E., J.W., M.T., J.S.). Activity 2 yielded brief, structured responses more suitable for descriptive content analysis. The analysis plan for each activity is detailed under the headings below.

#### Warm-up activity

Quantitative data were cleaned prior to analysis to ensure consistency. Responses given as a range (e.g. ‘20%–30%’) were standardized by taking the mid-point (e.g. 25%). Descriptive statistics were calculated, with the mean and standard deviation describing central tendency and variability and the median accounting for skewed distributions. Qualitative data from the warm-up activity are not presented in this paper.

#### Activity 1: World Café

Inductive thematic analysis was conducted following the six-phase framework outlined by Braun and Clarke (2006) to identify patterns related to how lived experience currently informs decision-making. Data were compared across the three decision-making settings, participant groups, and by the perceived effectiveness of involvement.

#### Activity 2: roundtables

Descriptive content analysis was used to analyse written responses generated during the roundtable discussions, drawing on approaches to qualitative content analysis described by Graneheim and Lundman (2004). Inductive coding was applied to categorize responses for each question. Category frequencies were calculated to indicate the relative prominence of participant responses. Categories were compared across the three decision-making settings to explore what makes some strategies more or less effective, how lived experience can be better combined with other types of evidence, and the tools required to implement change.

## Results

### Workshop participants

Eighty-nine summit attendees participated in the workshop. Of these, 36 (40.4%) selected at least one demographic group: 20.2% identified as LGBTIQA+, 13.5% reported living with a disability, 9.0% identified as Aboriginal and Torres Strait Islander, and 7.9% identified as culturally and linguistically diverse. Demographic information reflects only participants who selected one or more categories; eight participants (9.0%) selected multiple demographic characteristics. Demographic data on participants’ primary experience were not collected.

### Warm-up activity: the extent to which lived experience informs decision-making

Participant estimates of the extent to which lived experience knowledge informs suicide prevention decision-making ranged from 0% to 60%. The mean estimate was 16.5% (SD = 0.13), with a median of 12.5%. Most participants (80.0%) perceived lived experience knowledge to be incorporated into fewer than a quarter of decisions, with 93.8% perceiving it to be incorporated into fewer than half.

### Activity 1: how lived experience currently informs decision-making

Four overarching themes were constructed through inductive thematic analysis to reflect participants’ descriptions of how lived experience currently informs decision-making: *forms of engagement*, *genuine engagement*, *disingenuous engagement*, and *no decision-making authority*.

#### Forms of engagement

Participants described many forms of engagement currently used to incorporate lived experience knowledge into decision-making processes. These included co-design, board representation, advisory groups, focus groups, forums, panels, lived experience networks, consultation, feedback, surveys, and user testing. Some forms of engagement, such as ‘co-design’, were more often associated with effective engagement, whereas others, such as ‘consultation’, were described as less effective. However, participants consistently emphasized that effectiveness depended more on how the engagement is conducted. For example, disingenuous co-design was considered ‘less effective’. Additionally, many participants categorized lower-order levels of engagement, such as consultation, as effective, ‘depending on how [it was] conducted’. There were no notable differences in the reported forms of engagement across the three decision-making settings.

#### Genuine engagement

Genuine engagement was consistently identified as a key feature of effective integration of lived experience knowledge in decision-making. Engagement was described as genuine and most effective when underpinned by clear processes, transparency, and meaningful involvement, with participants emphasizing engagement being ‘authentic and transparent’, ‘well organized’, and ‘part of each stage’.

This theme was most prominent in discussions related to policy and strategy, and less frequently described in service design and delivery, and funding and priority-setting environments. Genuine engagement was raised by all groups, being most emphasized by people working in suicide prevention.

#### Disingenuous engagement

Disingenuous engagement was consistently described as ineffective. Participants highlighted several ways in which lived experience knowledge is used disingenuously in decision-making, most commonly through tokenistic practices, questioning whether lived experience knowledge was genuinely used or whether engagement processes were largely performative.

Sometimes it feels tokenistic so I wonder if it really is used to inform policy.

Tokenistic KPI meeting, box ticking, meeting funding agreements just for the $$.

Other descriptions outlined how engagement is often incomplete, under-prioritized, unpaid, or exploring only a limited diversity of experiences. Participants described that often people with lived experience ‘have to fight to be included’ and other ways engagement was disingenuous:

When lived experience isn’t involved in decision-making processes from start to finish.

Not effective [because] not meaningfully consulting the entire diverse range of [people with lived experience] (especially in marginalised, less privileged intersections).

While disingenuous engagement was noted across all three decision-making settings, it was most prominent in the policy and strategy setting. The theme was raised across participant groups but was most strongly emphasized by people with lived experience.

#### No decision-making authority

Participants described lived experience knowledge as being limited or absent from decision-making processes. Many reported minimal opportunities to contribute meaningfully, noting that there are ‘not many ways… would have liked to be asked, listened to, [and] heard’. Others were unequivocal in their views that people with lived experience do not currently inform decision-making, stating ‘they aren’t’, ‘not at all’, and ‘it isn’t’.

Perceptions of decision-making authority varied across settings and participant groups. Accounts of *no involvement* were most prominent in discussions related to funding and priority-setting, with related concerns being strongly emphasized by people working in suicide prevention and less so by other groups. In contrast, concerns of *limited involvement* were more salient among people with lived experience of suicidal distress and by family, friends, carers, and people bereaved by suicide.

### Activity 2: combining lived experience with other types of knowledge

Activity 2 focused on identifying and prioritizing strategies, challenges, and resource needs for integrating lived experience with other types of knowledge across decision-making settings. Results are presented following descriptive content analysis.

#### What makes some strategies more or less effective?

Four categories of effective strategies for integrating lived experience were generated through content analysis: meaningful engagement, structural enablers, sustainable and equitable resourcing, and building capacity. Three categories were also generated through content analysis to describe less effective strategies: superficial engagement, structural and political barriers, and unsustainable funding. The frequency and descriptions of categories are presented in Table 1.

**Table 1:** Content analysis of strategies for incorporating lived experience into decision-making.

Category	Description	%
What makes some strategies more effective?		
Meaningful engagement	Engagement that is clear, inclusive, equity-focused, and sustained throughout decision-making processes.	28.6%
Structural enablers	Shared leadership, accountability, and allowing sufficient time for involvement.	12.9%
Sustainable and equitable resourcing	Sustainable and equitable resourcing, with funding models that support the right organizations and prioritize lived experience leadership.	11.4%
Building capacity	Strengthening the lived experience workforce through skills development, training, and access to appropriate resources.	11.4%
What makes some strategies less effective?		
Superficial engagement	Engagement that is tokenistic, lacks diversity, is poorly implemented, or under-prioritizes lived experience.	20.0%
Structural and political barriers	Structural and political barriers, including changing priorities, siloed systems, and fragmented leadership.	12.9%
Unsustainable funding	Funding that is unsustainable.	2.9%

#### How can we better combine lived experience and other types of evidence to make better decisions in the future?

Two overarching categories were identified: *shifting power and recognizing diverse ways of knowing* (65.8%) and *cultivating cultures of openness, trust, and inclusivity* (34.2%). Sub-categories, descriptions, and exemplar quotes are summarized in Table 2.

**Table 2:** Content analyses of ways to better combine lived experience and other types of evidence.

Category	Sub-category	Description	Exemplar quotes
Shifting power and recognizing diverse ways of knowing (65.8%)	Sharing power (36.0%)	Redistributing or sharing power to increase decision-making authority for people with lived experience.	‘Power needs to be ceded or shared’ ‘Remunerated and supported [leadership positions]’
	Narratives and stories as evidence (28.0%)	The value of recognizing lived experience narratives and stories as legitimate forms of evidence.	‘Combine hard data with LE narratives’ ‘There is no book that can generate enough information [to equal] lived experience.’
	Challenging knowledge hierarchies (24.0%)	Challenging existing knowledge and decision-making hierarchies.	‘There is no credence given to experience’ ‘non-hierarchical collaboration’
	Maintaining a lived experience lens on research (12.0%)	Maintaining a lived experience lens throughout research and evaluation processes.	‘Evidence comes from the people—disconnect can come from relying on academia without connecting’
Cultivating cultures of openness, trust, and inclusivity (34.2%)	Mechanisms for engagement (38.5%)	The need for structured, early, and supported engagement mechanisms to embed lived experience meaningfully.	‘Early inclusion in the project so there is no pushback/ambiguity’ ‘Embedding LE co-design/consultation’
	Openness and adaptability (30.8%)	The importance of the suicide prevention sector adopting a mindset that embraces feedback and continuous improvement.	‘[being] willing to [receive] feedback + review what does/doesn’t work’ ‘Acknowledge existing research. Don’t be afraid to build on existing [research]’
	Trust and accountability (30.8%)	The need to integrate lived experience and community consultation into evidence-based practices to promote trust and establish accountability.	‘Adding appropriate community consultation into levels of evidence and critical appraisal tools as a ‘must have’ piece of the work being submitted to journals’

#### What tools or resources would be needed to improve evidence-informed practice?

Four categories were identified: *enabling inclusive and empowered decision-making*, *structural supports*, *a paradigm shift*, and *strengthening capacity for effective implementation*. Although participants were asked to identify tools and resources, many responses focused on broader organizational, structural, and cultural conditions required to support evidence-informed practice. As such, the findings include both tangible supports and wider enabling conditions considered necessary for meaningful integration of lived experience into decision-making. A narrative summary of each category is provided below.

*Enabling inclusive and empowered decision-making.* Tools that enable inclusive and empowered decision-making were most frequently identified (38.6%). Participants emphasized the importance of diverse representation in decision-making processes, including people from diverse backgrounds, underrepresented groups, and non-traditional suicide prevention sectors. Empowering lived experience expertise was also highlighted, including recognizing people with lived experience as experts and creating structures that support leadership roles and power-sharing.

Participants further stressed the importance of effective engagement and feedback mechanisms, including co-defining problems, addressing tokenism, and ensuring contributions can meaningfully inform decisions. Leadership buy-in was identified as critical, with participants noting that sustained commitment from organizational and system leaders is necessary to meaningfully and sustainably embed lived experience in decision-making. Participants also highlighted the importance of lived experience-informed evaluation, suggesting that appraisal and success measures should be shaped by lived experience.

*Structural supports*. Structural supports for integrating lived experience knowledge into decision-making were also commonly identified (22.7%). Participants described the need for fair compensation, strengthened governance arrangements, and adequate time and funding to meaningfully incorporate lived experience into decision-making processes.

*Paradigm shift.* Participants identified a need for a broader paradigm shift in how lived experience is valued and integrated into decision-making (20.5%). This included challenging traditional evidence hierarchies and ensuring knowledge generated through qualitative methods is equally recognized as valid evidence. Participants emphasized the need to address power dynamics, including the importance of ‘peer mutuality’ between people with lived experience and others.

A ‘reality check’ was also described as necessary to raise awareness of the importance of grounding decisions in real-life experiences. ‘Risk takers’ and ‘agitators’, who are willing to challenge the status quo, were identified as important drivers of cultural and systemic change.

*Strengthening capacity for effective implementation*. Strengthening the suicide prevention sector’s capacity for effective implementation was also highlighted (18.2%). Participants described the need for capacity building not only within the suicide prevention workforce but also among people with lived experience.

Communities of practice were identified as valuable mechanisms for ongoing peer learning and shared problem-solving. Participants also emphasized the importance of improving the implementation of existing approaches, noting that effective ideas and frameworks already exist. Rather than developing new approaches, participants suggested greater attention should be directed towards strengthening implementation in practice. Finally, participants identified the need for critical appraisal tools or frameworks to assess whether lived experience perspectives genuinely shape decisions or are merely tokenistic.

## Discussion

This study provides insight into how lived experience currently informs decision-making in suicide prevention, how different forms of evidence can be reconciled, and what is required to strengthen the integration of lived experience into decision-making processes. Consistent with the study aims, the discussion is organized around three key findings relating to (i) current approaches to lived experience involvement; (ii) how lived experience knowledge is positioned alongside other forms of evidence; and (iii) the tools and resources required to strengthen evidence-based decision-making in suicide prevention.

While there are many ways lived experience is currently meaningfully integrated into suicide prevention decision-making processes, disingenuous engagement remains prevalent. Most participants reported that lived experience was only involved in a small proportion of decisions. While levels of engagement (e.g. consultation vs. co-design) influenced perceived effectiveness, the genuineness of engagement was identified as an overall stronger determinant. Genuine engagement was characterized by clear scope and purpose, transparency, appropriate resourcing, diverse representation, fair compensation, and integration across all stages of decision-making. In contrast, ineffective approaches were tokenistic, limited in diversity, under-resourced, non-existent, or confined to isolated stages of the process. These findings align with participation frameworks that associate higher levels of engagement, such as collaboration and empowerment, with more meaningful integration of lived experience (International Association for Public Participation 2018, Roses in the Ocean 2022a), and extend criticisms of ‘faux-design’, where processes labelled as co-design are tokenistic and fail to result in genuine engagement (Oldman *et al*. 2025). Faux-design is known to lead to frustration, reduced trust, and disengagement, all of which are detrimental to embedding lived experience into decision-making processes (Carman *et al*. 2013, Commonwealth of Australia 2017, Madden and Speed 2017, Oldman *et al*. 2025). The findings highlight that lived experience involvement alone does not guarantee influence. It is essential for lived experience engagement to extend beyond rhetoric and performativity. Instead, engagement must be meaningful and grounded in the redistribution of power within decision-making processes. This distinction is particularly relevant in funding and priority-setting contexts, where participants most frequently reported perceptions of exclusion.

The second key finding relates to systemic issues of power and sector culture and how lived experience knowledge is valued and reconciled alongside other forms of evidence. Participants consistently identified the need for broader cultural and structural change, with the redistribution of power and decision-making authority to people with lived experience as central to this. As a sector, this requires moving from conceptualizing lived experience engagement as an aspirational gold standard to embedding it as standard practice. Accountability mechanisms are required to ensure that minimum standards are met. For instance, the National Suicide Prevention Strategy Outcomes Map links the National Suicide Prevention Strategy 2025–2035 outcomes, including the critical enabler ‘embedded lived experience’, to indicators and data measures to monitor change nationally (National Suicide Prevention Office 2026). Developed in partnership with the National Suicide Prevention Office Lived Experience Partnership Group, the National Suicide Prevention Outcomes Framework and Map provide a shared understanding of how lived experience can be measured, while also acting as a precedent for how people with lived experience can be incorporated into decision-making processes (National Suicide Prevention Office 2026). Other accountability mechanisms, such as emerging reporting requirements in academic publishing (Davis *et al*. 2024), also demonstrate formalizing expectations and shifting lived experience involvement from symbolic inclusion towards demonstrable influence on decisions. However, accountability mechanisms alone are insufficient. Adequate and equitable funding and resourcing are also necessary to enable programmes and services to meet minimum standards.

These findings highlight how dominant evidence hierarchies can privilege certain forms of knowledge, particularly quantitative approaches, while qualitative and lived experience knowledge is often positioned as supplementary rather than constitutive of evidence (Luke *et al*. 2022, Roses in the Ocean 2025). Such framings risk marginalizing experiential knowledge and limiting its influence on decisions. Strengthening how lived experience is integrated into evidence-based decision-making requires a systemic shift in how evidence is defined, operationalized, and implemented in suicide prevention. This study contributes insight into how these hierarchies operate within suicide prevention decision-making settings, extending transdisciplinary models of evidence-based practice by demonstrating how lived experience is negotiated alongside other forms of evidence in practice.

Lastly, this study identifies practical tools and resources required to strengthen lived experience involvement in evidence-based decisions in suicide prevention. Notably, when asked about tools and resources, participants frequently identified broader organizational and structural conditions that would enable effective involvement, suggesting that improving evidence-informed practice requires attention to the broader system enablers as well as tangible resources. The practical and system tools identified in the research can be categorized into four key areas of focus. First, practical guidance is needed to support individuals and organizations to meaningfully embed lived experience into suicide prevention approaches, including a clear articulation of what constitutes genuine engagement and indicators of tokenistic or ‘faux-design’ practice. Second, tools are required to guide the structural supports that underpin meaningful engagement, such as governance arrangements, appropriate compensation, funding allocation, and time considerations. Greater clarity is needed for both funders and programme and service providers to ensure that planned lived experience engagement is proportionate and appropriately resourced and avoids rhetorical and tokenistic practices. Third, continued advocacy is required to support broader cultural and systemic change across the sector regarding how lived experience is valued and empowered. Finally, strengthening implementation capacity is critical to ensuring lived experience engagement is effectively enacted in practice. Several tools and resources already exist to support meaningful engagement; however, participants’ responses suggest that the existence of resources alone is insufficient. Effective implementation also requires leadership commitment, supportive governance arrangements, appropriate resourcing, capability development, and cultural change. It is therefore essential to avoid duplication and instead focus on both implementation of existing guidance and the broader conditions needed for its successful application across the sector.

### Lived and living experience reflections on the research

The reflections below provide lived and living experience interpretations of the findings, highlighting how issues of power, representation, and accountability are experienced in practice, as well as what is needed to better integrate lived experience into evidence-based decision-making in suicide prevention.

#### Meaningful engagement, power, and representation

Engaging individuals with a lived and living experience of suicide requires a foundation of safety, equity, and integrity. Meaningful participation goes beyond initial engagement; ongoing involvement deepens understandings of identities, environments, and experiences and strengthens the relevance of decision-making.

There is a widely held view that a select cohort of people with lived and living experience sit on multiple advisory boards and are given more opportunities to influence decisions. While their contributions are valuable, they do not represent the full diversity of experiences. Increased support and investment are needed to enable more diverse voices and more people to participate and shape decisions.

Engagement should always recognize that people’s experiences, time, skills, and emotional investment are deserving of acknowledgement and fair remuneration. This is vital to systemic reform, service implementation, and advocacy. Without clear, transparent, and consistent payment guidelines from organizations, people with lived and living experience may have the belief they do not need or deserve recognition. This is especially true if they are not well-established in the sector, confident in their skills, or understand they have the right to be compensated.

Tokenistic engagement and box ticking damage relationships. This not only undermines confidence in those wishing to participate but also cheapens the vital contribution that people with lived and living experience have to offer. People with lived and living experience are powerful change makers, not a KPI and deserve to participate in suicide prevention decision-making processes. Genuine collaboration would go a long way towards putting the control and direction of suicide prevention into the hands of those most affected.

#### What is needed?

Utilizing lived and living experience and collaborative approaches with researchers, academics, and organizations requires clear accountability, shared values, autonomy, and transparency. Building capacity and capabilities of those with lived experience in suicide prevention demands careful formation, flexibility, and ethically bound frameworks. Workshop facilitation, co-design, or collaborative projects must be undertaken with consistent access, standards, and engagement approaches.

Better education will support engagement techniques, including assisting people newer to the sector to understand existing structures and frameworks, as well as training that enables people with lived and living experience to explore their strengths, how to use their experience, and possible career pathways. Education must also extend to the existing sector, allowing for further development of the lived and living experience workforce, especially into leadership roles. People wishing to step up must be supported to understand the existing landscape, including all its complexities and funding structures. Education must be funded to support people from all areas of the sector, from geographic location, and from small grassroots to larger organizations.

Knowledge is powerful, and increasing education will help to address existing power imbalances while supporting greater diversity within the sector, something that is desperately needed for continued growth and development.

Alongside the need for greater education and workforce development, questions remain about how people’s engagement and advocacy is fairly and consistently recognized at a national level. From navigating the sector as people with lived and living experience, existing guidelines for remuneration have been shown to have inconsistent payment structures, reimbursement standards, and eligible activities. Consistent standards will protect the experiences, journeys, and skills of people with lived and living experience across government, non-government, not-for-profit, and grassroots organizations.

We have made significant progress towards integrating lived and living experience into suicide prevention decision-making. With better education, funding, and genuine collaboration, the Australian suicide prevention sector can continue to lead the way. We owe it to those struggling, those lost, and those we will lose.

### Strengths and limitations

Several strengths and limitations should be considered when interpreting these findings. Participants self-selected to attend the conference and the workshop and may have been more supportive of lived experience involvement in decision-making than the broader suicide prevention sector. Attendance also required time and financial commitments, potentially limiting participation for individuals without organizational support or the required resources. While it is common for conference workshops to bring together diverse participants to collaborate and generate new ideas, future research should consider the ethics of conducting research in settings where participants, particularly those with lived experience, have paid to attend. The conceptualization of this research, design and delivery of the workshop, and interpretation of findings were strengthened by people with lived experience.

## Conclusion

While lived experience is increasingly recognized in suicide prevention, its integration into evidence-based decision-making remains inconsistent and constrained by entrenched power imbalances and knowledge hierarchies. Improving policy and practice requires more than additional resources to guide lived experience engagement; it demands a paradigm shift that positions lived experience knowledge as equal to, and fully embedded within evidence, rather than oppositional to it.

## Data Availability

Due to the nature of the research and ethical considerations relating to participant privacy and confidentiality, the supporting data are not available.
